# The Effects of Meteorological Factors on Dengue Cases in Malaysia

**DOI:** 10.3390/ijerph19116449

**Published:** 2022-05-26

**Authors:** Sarbhan Singh, Lai Chee Herng, Lokman Hakim Sulaiman, Shew Fung Wong, Jenarun Jelip, Norhayati Mokhtar, Quillon Harpham, Gina Tsarouchi, Balvinder Singh Gill

**Affiliations:** 1 Institute for Medical Research, Ministry of Health, Shah Alam 40170, Malaysia; jochua0505@gmail.com (L.C.H.); drbsgill@moh.gov.my (B.S.G.); 2 Institute for Research, Development and Innovation (IRDI), International Medical University, Kuala Lumpur 57000, Malaysia; lokmanhakim@imu.edu.my; 3 School of Medicine, International Medical University, Kuala Lumpur 57000, Malaysia; shewfung_wong@imu.edu.my; 4 Centre for Environmental and Population Health, Institute for Research, Development and Innovation (IRDI), International Medical University, Kuala Lumpur 57000, Malaysia; 5 Vector Borne Disease Control Division, Ministry of Health Malaysia, Putrajaya 62000, Malaysia; jenarun@moh.gov.my (J.J.); drnorhayati_m@moh.gov.my (N.M.); 6 HR Wallingford, Wallingford OX10 8BA, UK; q.harpham@hrwallingford.com (Q.H.); g.tsarouchi@hrwallingford.com (G.T.)

**Keywords:** dengue, rainfall, temperature, wind speed, earth observation

## Abstract

Dengue is a vector-borne disease affected by meteorological factors and is commonly recorded from ground stations. Data from ground station have limited spatial representation and accuracy, which can be overcome using satellite-based Earth Observation (EO) recordings instead. EO-based meteorological recordings can help to provide a better understanding of the correlations between meteorological variables and dengue cases. This paper aimed to first validate the satellite-based (EO) data of temperature, wind speed, and rainfall using ground station data. Subsequently, we aimed to determine if the spatially matched EO data correlated with dengue fever cases from 2011 to 2019 in Malaysia. EO data were spatially matched with the data from four ground stations located at states and districts in the central (Selangor, Petaling) and east coast (Kelantan, Kota Baharu) geographical regions of Peninsular Malaysia. Spearman’s rank-order correlation coefficient (ρ) was performed to examine the correlation between EO and ground station data. A cross-correlation analysis with an eight-week lag period was performed to examine the magnitude of correlation between EO data and dengue case across the three time periods (2011–2019, 2015–2019, 2011–2014). The highest correlation between the ground-based stations and corresponding EO data were reported for temperature (mean ρ = 0.779), followed by rainfall (mean ρ = 0.687) and wind speed (mean ρ = 0.639). Overall, positive correlations were observed between weekly dengue cases and rainfall for Selangor and Petaling across all time periods with significant correlations being observed for the period from 2011 to 2019 and 2015 to 2019. In addition, positive significant correlations were also observed between weekly dengue cases and temperature for Kelantan and Kota Baharu across all time periods, while negative significant correlations between weekly dengue cases and temperature were observed in Selangor and Petaling across all time periods. Overall negative correlations were observed between weekly dengue cases and wind speed in all areas from 2011 to 2019 and 2015 to 2019, with significant correlations being observed for the period from 2015 to 2019. EO-derived meteorological variables explained 48.2% of the variation in dengue cases in Selangor. Moderate to strong correlations were observed between meteorological variables recorded from EO data derived from satellites and ground stations, thereby justifying the use of EO data as a viable alternative to ground stations for recording meteorological variables. Both rainfall and temperature were found to be positively correlated with weekly dengue cases; however, wind speed was negatively correlated with dengue cases.

## 1. Introduction

Dengue is an Arboviral disease that is spreading rapidly across tropical and subtropical regions throughout the world [1]. With the global incidence of dengue infections rising dramatically at rates of 250 million infections each year in recent decades, it is concerning that now about half of the world’s population is at risk of contracting dengue [2]. The *Aedes aegypti* and *Aedes albopictus* mosquitoes are the primary vectors that are responsible for the transmission of the dengue virus to humans [1]. Dengue transmission is affected by several interrelated factors such as meteorological, hydrological and social factors and virus serotypes [3]. Meteorological variables such as rainfall, temperature and humidity are weather factors strongly associated with dengue outbreaks. For example, increased rainfall and temperature were shown to increase the incidence of dengue infections, by increasing the number of mosquito breeding sites, enhancing the mosquito breeding cycle, promoting vector development (maturation period shortened), increasing vector survival and blood-feeding patterns. This results in an increase in the vector population and subsequently increases the probability of vector–human interaction, which leads to increased viral transmission [4,5].

In Malaysia, a recent systematic review by Hii et al. (2016) identified several studies that reported on the relationship between various meteorological factors and dengue infections [6]. Most of these studies reported that temperature, rainfall, and wind speed influence the dynamics of dengue disease. However, the majority of these studies used meteorological data recorded from ground-based stations to examine the relationship of weather variables with dengue infections [6]. Meteorological data recorded at ground-based stations are easily accessible, have a low cost, and may be considered the gold standard as per the World Meteorological Organization standards for obtaining meteorological data [7,8]. 

However, using meteorological data from ground-based stations may not provide an accurate representation of weather data in a given area as they are subject to several limitations [7]. These include the fact that ground-based stations are more spatially representative of certain areas of interest rather than others. For example, ground-based stations tend to be centred around locations that have key industries of interest (i.e., airports) [7]. Therefore, accurate and reliable meteorological data are often not available for areas that lie at greater distances beyond the coverage of ground-based stations. Other limitations include the availability of only the point reading of weather data from ground stations and failure to maintain complete records, resulting in missing data [7].

To overcome these limitations, several studies suggested the use of Earth Observation (EO) meteorological data derived from satellites as an alternative for meteorological data recorded at ground-based stations [7]. This is because EO data obtained from satellites tend to have a wide coverage which could be used to obtain meteorological data in areas where ground-based measurements are unavailable or incomplete. Several studies have reported on the validity of EO meteorological data against data recorded at ground-based stations [7,9,10], wherein the majority of these studies conclude that EO data are valid and reliable to use. As each of the above studies derives EO data from a range of satellites, it is important to validate the EO data for each study location.

More recently, EO data derived from satellites has been used in several dengue-related studies [11,12]. However, as of yet, in Malaysia, no published studies exist that use EO meteorological data to examine the correlation between temperature, wind speed, and rainfall with dengue infections. With the existing limitations of ground-based meteorological data, it is important to ensure that EO data derived from satellites are well correlated with spatially matched ground station data to justify the use of EO weather data as an alternative to ground station data. In this study, we first validated the satellite-based EO data observations of (temperature, wind speed, and rainfall) with ground station data. Subsequently, we determined if the spatially matched EO data correlates with dengue cases from 2011 to 2019 in Malaysia. 

## 2. Materials and Methods

### 2.1. Study Location

The data were obtained from states and districts in the central (Selangor, Petaling) and east coast (Kelantan, Kota Baharu) geographical regions of Peninsular Malaysia. These areas were selected for the following reasons: (a) The variation in climatic patterns between the east and west coast of Peninsular Malaysia, wherein the North East Monsoon (NEM) affects states in the east coast of Peninsular Malaysia (i.e., Kelantan) from November to March, while the South West Monsoon (SWM) affects states in the central south west region of Peninsular Malaysia (i.e., Selangor) from May to September [13]; (b) both Selangor and Kelantan reported the highest number of dengue infectious within the respective Southwest and Northeast geographical regions of Peninsular Malaysia from 2011 to 2019 as shown in Appendix A—Figure A1 [14]; (c) all selected states had a high population density [15].

### 2.2. Study Data 

The following three types of data were used in this study: (a) EO-based meteorological data, (b) ground-based station meteorological data, and (c) dengue case data. The data used in this study are open-source data and were obtained for the period from January 2011 to December 2019.

(a) EO-based meteorological data

**Figure 1:**
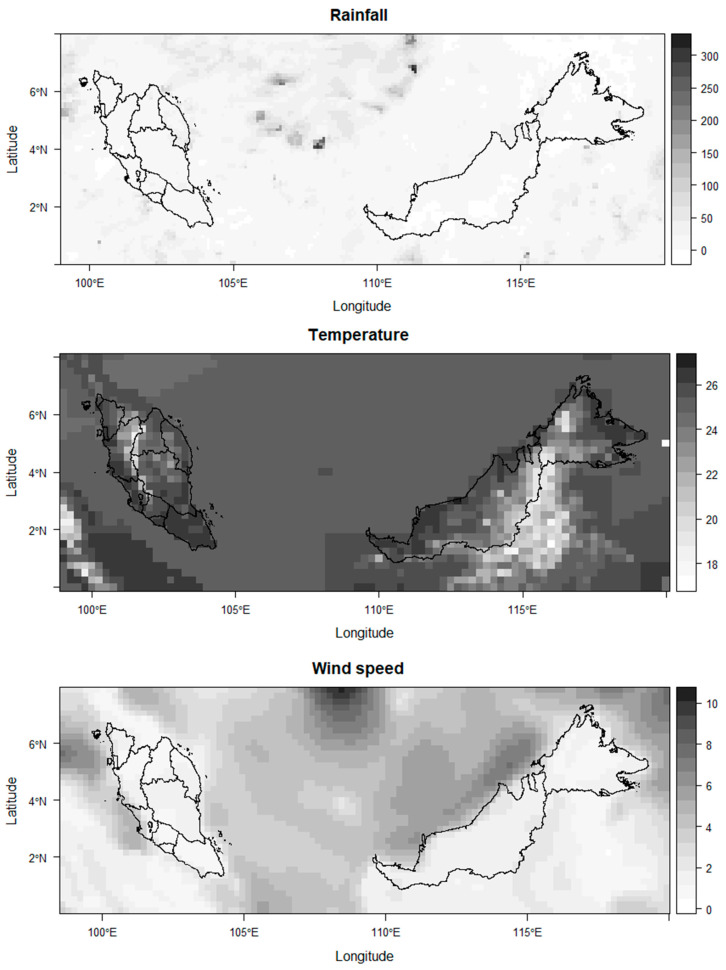
Raster surface for EO meteorological variables for 1 April 2019, Peninsular Malaysia.

Rainfall data were sourced from the Global Precipitation Measurement (GPM) and Tropical Rainfall Measuring Mission (TRMM) [16]. Temperature data were sourced from the MODIS Terra and Aqua—Modis NASA Distributed Active Archive Center (DAAC)), which are sources of land surface temperatures (i.e., maximum, minimum, and mean temperature), and were converted to land air temperatures [17]. Wind speed was sourced from the Climate Forecast System version 2 (CFSv2) of the National Centres for Environmental Prediction (NCEP) [18]. Further information on the EO-based data is shown in Table 1. The EO meteorological data were obtained in raster format, with a spatial extent of latitude from 00 to 80 N and longitude from 98.50 E to 1200 E. The spatial resolution for rainfall was 0.1° × 0.1° longitude–latitude grid; for temperature it was 0.05° × 0.05°; and for wind speed it was 0.205° × 0.205°, as shown in Figure 1. The temporal resolution of the EO variables was measured daily.

(b) Ground-based station meteorological data

**Figure 2:**
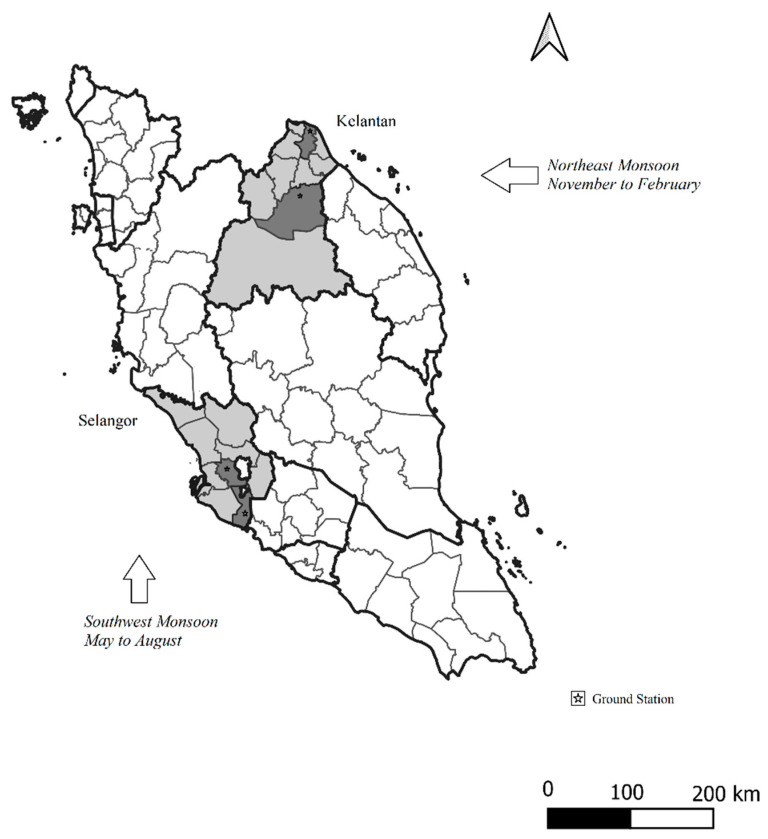
Rainfall, Temperature and Wind speed data collected from Ground based stations (*n* = 4) in Peninsular Malaysia.

The ground station data for Malaysia were sourced from the National Oceanic and Atmospheric Administration (NOAA) and are available at https://www.noaa.gov/ (open source) for a duration of nine years (1 January 2011 to 31 December 2019). A total of four ground stations were included in this study namely, the Kota Baharu ground station (Station ID 486150, latitude 6.170, longitude 102.290), Kuala Krai ground station (Station ID 48616, latitude 5.530, longitude 102.200), Subang Jaya ground station (Station ID 486470, latitude 3.130, longitude 101.550), and Sepang ground station (Station ID 486500, latitude 2.740, longitude 101.710) which provided data on rainfall, temperature, and wind speed (ground station locations are shown in Figure 2). The stations provided daily values for the period from January 2011 to December 2019 (same with the EO data). The ground-based stations used the same units of measurement with the EO data except for temperature which was measured in Fahrenheit (°F) and converted to °C.

(c) Dengue case data

Dengue case data from 2011 to 2019 were sourced from the dengue surveillance database of the Disease Control Division, Ministry of Health, Malaysia. Since the implementation of the Prevention and Control of Infectious Disease Act 1988 (Act 342) in Malaysia, it has been mandatory to report all laboratory-confirmed dengue cases within 24 h to the nearest health officer [19]. A confirmed dengue case is one that meets the clinical case definition and laboratory confirmation of dengue fever [19]. Daily dengue data were aggregated based on weeks (based on epidemiology weeks, which start on Sunday) and according to states and districts in Malaysia [20]. The data set comprised of aggregated data with all personal identifiers removed. The spatial distribution of the dengue cases is represented at the level of districts and states.

### 2.3. Data Analysis

Data were analysed using the R programming software version 4.0.3 by Hornik and R Core Team, RStudio, PBC from 250 Northern Ave, Boston, MA, United States of America and Statistical Package for the Social Sciences (SPSS) version 26.0 by the International Business Machines, IBM Corp. Released 2019 from Armonk, NY, United States of America. Before the analysis, data pre-processing for the dengue case and meteorological data included checking for missing values, duplicate values, abnormal values and outliers. Both the dengue case data and ground station-based meteorological data were valid, complete, and free from missing, duplicate or abnormal values. The EO-based meteorological data were cleaned, while missing values which constituted 0.5% were omitted from the analysis and duplicate entries which constituted 0.2% were removed (Appendix A—Figure A2). The analysis was conducted by aggregating the daily data into weeks, which was based on epidemiological weeks (with Epid week beginning from Sunday) [20]. Weekly rainfall was represented by a 7-day cumulative rainfall in a given week. Weekly temperature and wind speed were represented by a 7-day average in a given week. The analyses performed were, first, a correlation analysis between the EO-based data (temperature, rainfall, and wind speed) and ground station meteorological data (temperature, rainfall, and wind speed). Second, a cross correlation analysis was performed with an 8-week lag period to examine the magnitude of correlation between the EO-based data (temperature, rainfall, and wind speed) and dengue infections. Third, a multivariate linear regression analysis was conducted that aims to examine the relationship between the meteorological variables (temperature, rainfall and wind speed) and dengue cases. Details of the analysis are further explained below.

#### 2.3.1. Correlation Analysis between EO-Based and Station-Based Meteorological Data 

Data were tested for normality prior to the correlation analysis using the Shapiro–Wilk test, wherein *p* values < 0.05 indicate a normal distribution [21]. Using the coordinates from the ground station, we determine the corresponding pixel from the raster file, from which the EO-based values were extracted. These values were used to correlate with the ground station data readings. A correlation analysis between the EO and ground-based station meteorological data of temperature, rainfall, and wind speed was performed using Spearman’s rank-order correlation coefficient (ρ) wherein ρ values between the ranges of less than 0.10, 0.10 to 0.39, 0.40 to 0.69, 0.70 to 0.89, and 0.90 to 1.00 indicated negligible, weak, moderate, strong, and very strong correlation, respectively [22]. The significance level of the correlation was set at *p* < 0.05. The correlation between the ground station and EO meteorological data was conducted from 2 January 2011 to 28 December 2019, and for a total of 468 weeks.

#### 2.3.2. Correlation Analysis between Meteorological Data and Dengue Cases

The correlation between EO-based data (temperature, rainfall, and wind speed) and dengue infections was examined using a cross-correlation analysis with an eight-week lag period to examine the magnitude of correlation. The cross-correlation was performed at the state and district levels, wherein dengue cases were represented by all cases within the specific state and district, respectively, from the following three time periods: (a) the overall period from 2011 to 2019, (b) 2011 to 2014 (prior to change in the dengue surveillance and testing process) and (c) 2015 to 2019 (after revisions to the dengue surveillance and testing process) [23]. In order to match the EO-based meteorological data and cases representative of a spatial area (state/district boundary), all cases for that area and all pixel values of EO data for the same area were used. The average value of each meteorological variable for each epidemiological week was estimated using a spatial extraction procedure which was performed for the respective states and districts. The spatial extraction was based on state and district polygon layers provided by Malaysian Centre for Geospatial Data Infrastructure (MaCGDI) and National Geospatial Centre (PGN). Spatial extraction (mathematical clipping) was performed using the R programming language with the aid of SP, raster, ncdf4 and rgdal base statistical and spatial processing packages. EO-based meteorological data were imported into the R programming software as a raster object and shapefiles that contained state and district administrative border polygons. Data were extracted from the raster file and subsequently aggregated based on mean values. Extracted data were then formatted and checked for completeness and duplicates prior to performing the cross-correlation analysis. 

#### 2.3.3. Multivariate Linear Regression Analysis between Meteorological Variables and Dengue Cases

A multivariate linear regression analysis was performed to examine the relationship between the meteorological variables (temperature, rainfall and wind speed) and dengue cases. Selangor state was selected for the multivariate linear regression analysis as it had the highest cross correlation values between meteorological variables and dengue cases in the cross correlation analysis. The assumption of multivariate linear regression were satisfied and were as follows: evidence of linearity and homoscedasticity was ascertained by the linear and homoscedastic distribution of the predicted and residual values on the scatter plot (Appendix A—Figure A5). There was no evidence of multicollinearity as the Variation Inflation Factor (VIF) values for the predictor variables ranged from 1.26 to 1.49 (less than 10). A log transformation was applied to the factor variable (dengue case) to satisfy the assumption of normality (Kolmogorov–Smirnov test of normality *p* value 0.200). The level of significance for the regression model was set at *p* < 0.05. Standardized Coefficients Beta (B) and R square was reported.

## 3. Results

### 3.1. Correlation between EO and Station-Based Meteorological Data 

**Figure 3:**
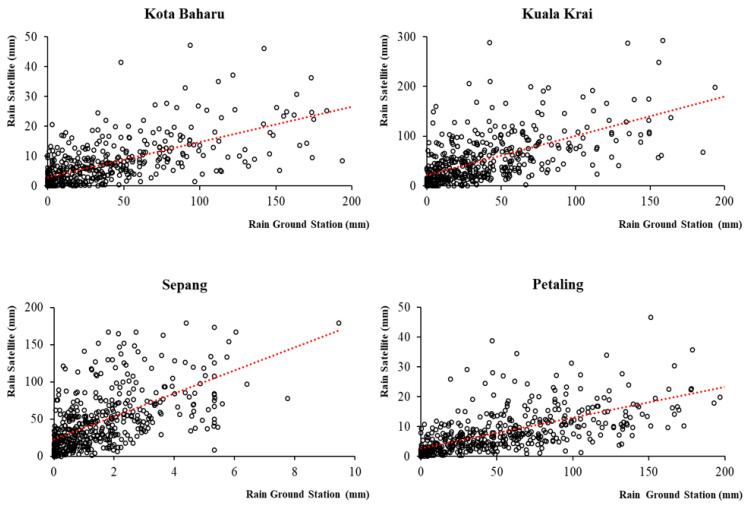
Correlation between rainfall data from EO and the four ground-based stations, 2011 to 2019, Malaysia.

**Figure 4:**
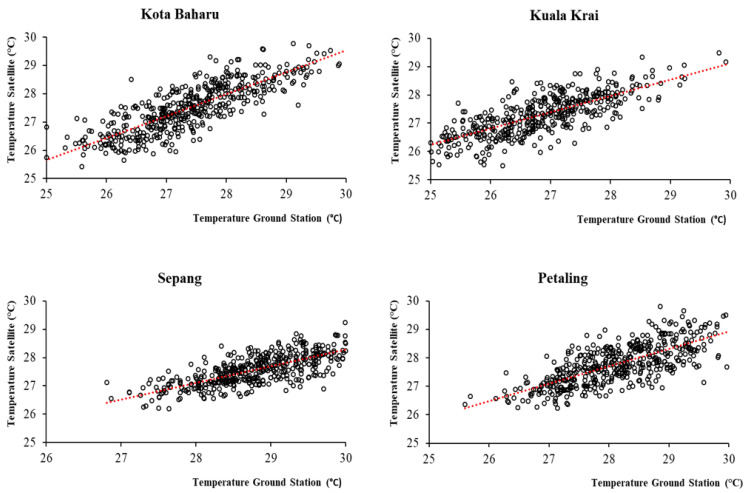
Correlation between temperature data from EO and the four ground-based stations, 2011 to 2019, Malaysia.

**Figure 5:**
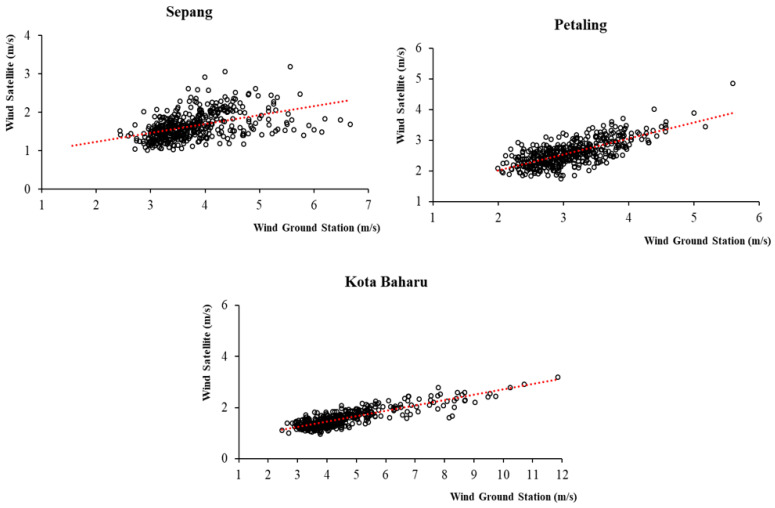
Correlation between wind speed data from EO and ground-based stations (*n* = 3), 2011 to 2019, Malaysia.

All the EO meteorological variables were found to be significantly positively correlated with the meteorological data recorded by the respective ground stations. Rainfall estimates were reported to have significant, positive and moderate to strong ρ correlations of 0.641, 0.669, 0.702 and 0.736 for the Sepang, Kuala Krai, Subang and Kota Baharu ground stations, respectively. Temperature estimates were found to have significant, positive and strong ρ correlations of 0.724, 0.757, 0.797 and 0.838 for the Subang, Sepang, Kuala Krai and Kota Baharu ground stations, respectively. Wind speed estimates were found to have significant, positive and moderate to strong ρ correlations of 0.519, 0.658 and 0.742 for the Sepang, Subang and Kota Baharu ground stations, respectively. Overall, the correlations across all the meteorological parameters were much stronger in the Kota Baharu district compared to the other districts. When comparing the magnitude of correlation among meteorological variables, the highest correlation between the ground-based stations and corresponding EO data were reported for temperature (mean ρ = 0.779), followed by rainfall (mean ρ = 0.687) and wind speed (mean ρ = 0.639). The correlation values for the meteorological data between the four ground-based stations and the corresponding EO data are shown in Table 2. Figure 3, Figure 4 and Figure 5, show the scatter plots for the correlations between ground-based stations and the corresponding EO data.

### 3.2. Cross-Correlation between EO Data (Temperature, Rainfall and Wind Speed) and Dengue Cases

(a) Rainfall and dengue cases

**Figure 6:**
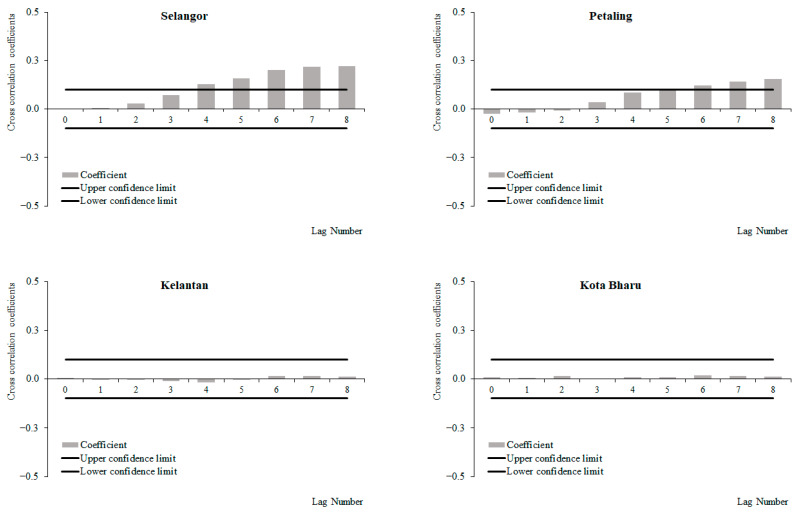
Cross-correlation function between dengue cases and rainfall in Selangor, Petaling, Kelantan and Kota Baharu, 2011 to 2019.

**Figure 7:**
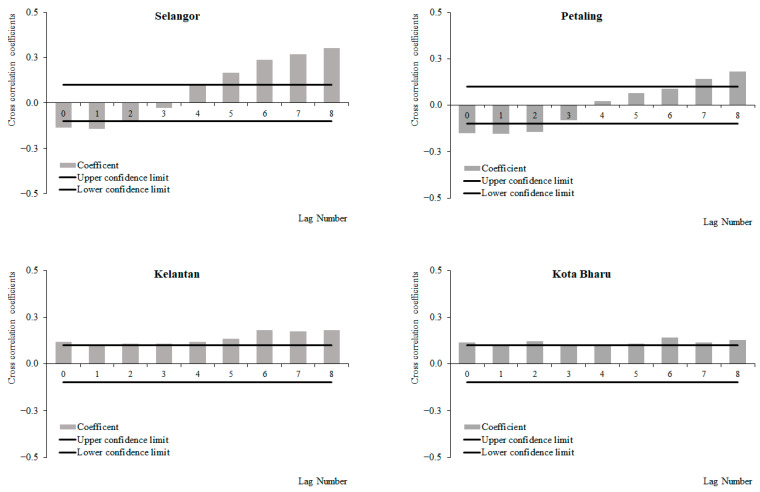
Cross-correlation function between dengue cases and rainfall in Selangor, Petaling, Kelantan and Kota Baharu, 2015 to 2019.

**Figure 8:**
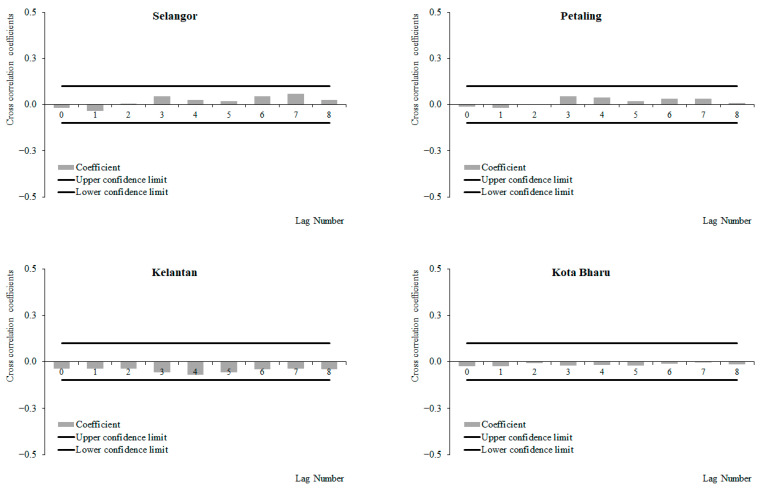
Cross-correlation function between dengue cases and rainfall in Selangor, Petaling, Kelantan and Kota Baharu, 2011 to 2014.

For the period from 2011 to 2019, a positive correlation between weekly dengue cases and rainfall at lags 2 and 3, which increased with subsequent lags and became significant at lags 4 and 6 onwards was observed for Selangor and Petaling, respectively (Figure 6). No significant correlations between weekly dengue cases and rainfall were identified in Kelantan and Kota Baharu for the period from 2011 to 2019. The highest correlation values were observed at lag 8 for Selangor (0.223) and Petaling (0.155) and lag 6 for Kelantan (0.015) and Kota Baharu (0.018). Meanwhile, for the period between 2015 and 2019, a positive correlation between weekly dengue cases and rainfall at lags 0 and 4 was observed, which increased with subsequent lags and became significant at lags 2, 4, 5, 7 and onwards for Selangor, Petaling, Kelantan and Kota Baharu, respectively (Figure 7). The highest correlation values were observed at lag 8 for Selangor (0.304), Petaling (0.181), Kelantan (0.181) and lag 6 for Kota Baharu (0.142). During the periods from 2011 to 2014, no significant correlations were reported between weekly dengue cases and rainfall (Figure 8). Overall positive correlations were observed between weekly dengue cases and rainfall for Selangor and Petaling across all the studied time periods with significant correlations observed for the periods from 2011 to 2019 and 2015 to 2019. In addition, all areas were found to have positive significant correlations between weekly dengue cases and rainfall for the period from 2015 to 2019.

(b) Temperature and dengue cases

**Figure 9:**
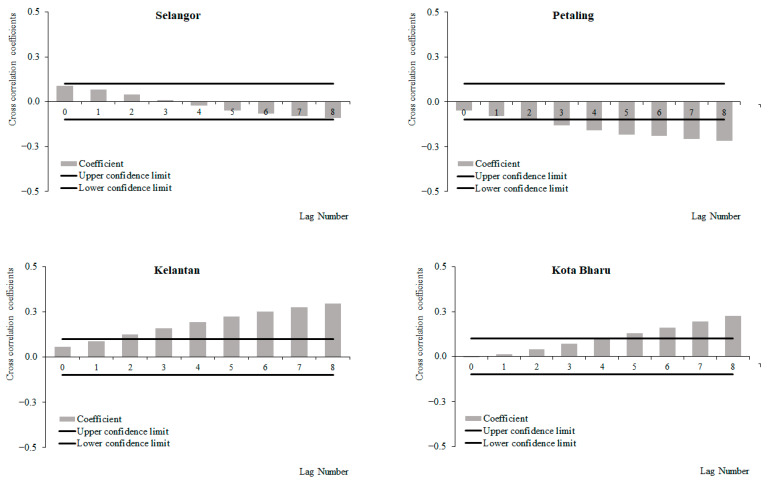
Cross-correlation function between dengue cases and temperature in Selangor, Petaling, Kelantan and Kota Baharu, 2011 to 2019.

**Figure 10:**
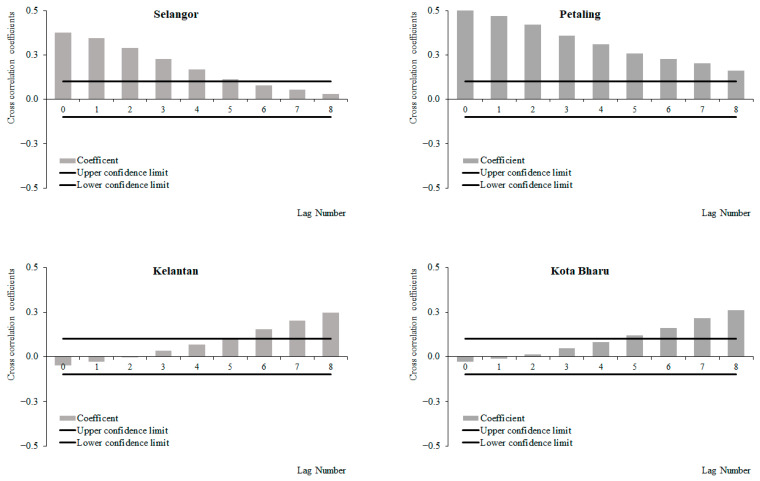
Cross-correlation function between dengue cases and temperature in Selangor, Petaling, Kelantan and Kota Baharu, 2015 to 2019.

**Figure 11:**
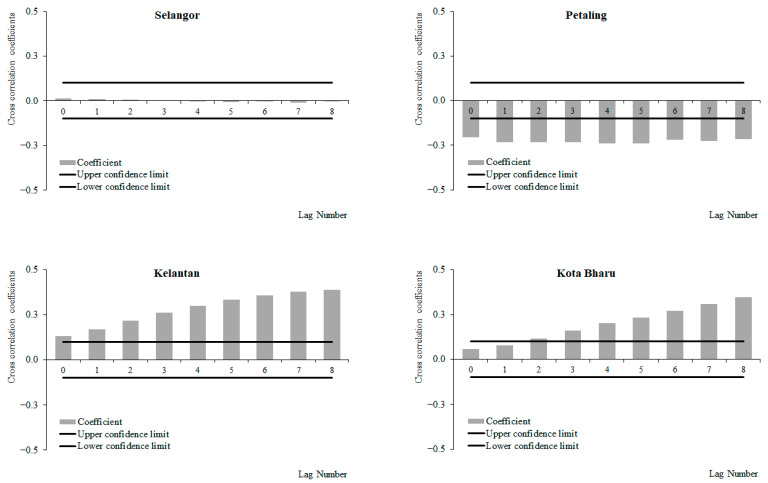
Cross-correlation function between dengue cases and temperature in Selangor, Petaling, Kelantan and Kota Baharu, 2011 to 2014.

For the period from 2011 to 2019, a negative correlation between weekly dengue cases and temperature at lags 0 and 4, which increased with subsequent lags was observed in Selangor and Petaling, respectively (Figure 9), with significant negative correlations at lag 3 onwards for Petaling. Meanwhile, a positive correlation between weekly dengue cases and temperature at lags 0 and 1 was observed, which increased with subsequent lags and became significant at lags 2 and 5 onwards in Kelantan and Kota Baharu. The highest correlation values were observed at lag 8 for Selangor (−0.010), Petaling (−0.217), Kelantan (0.294) and Kota Baharu (0.227), respectively. For the period from 2015 to 2019, positive correlations between weekly dengue cases and temperature at lags 0, 2 and 3 were observed, which decreased (i.e., for Selangor and Petaling) and increased (i.e., Kelantan and Kota Baharu) with subsequent lags with significant positive correlations at lag 0, 5 and 6 onwards for Selangor, Petaling, Kota Baharu and Kelantan, respectively (Figure 10). The highest correlation values were observed at lag 0 for Selangor (0.376), Petaling (0.511), and lag 8 for Kelantan (0.247) and Kota Baharu (0.260), respectively. During the periods between 2011 and 2014, positive correlations between weekly dengue cases and temperature at lag 0, which increased with subsequent lags, with significant positive correlations at lags 0 and 2 onwards were observed for Kelantan and Kota Baharu (Figure 11). Conversely, a negative correlation between weekly dengue cases and temperature at lags 0 and 5, which increased with subsequent lags, was observed in Selangor and Petaling, with significant negative correlations at lag 0 onwards for Petaling. The highest correlation values were observed at lag 8 for Kelantan (0.388) and Kota Baharu (0.346), lag 4 for Petaling (−0.240) and lag 7 for Selangor (−0.010). 

Overall positive significant correlations were observed between weekly dengue cases and temperature for Kelantan and Kota Baharu across all time periods. Negative/reduced positive significant correlations between weekly dengue cases and temperature were observed in Selangor and Petaling across all time periods, respectively.

(c) Wind speed and dengue cases

**Figure 12:**
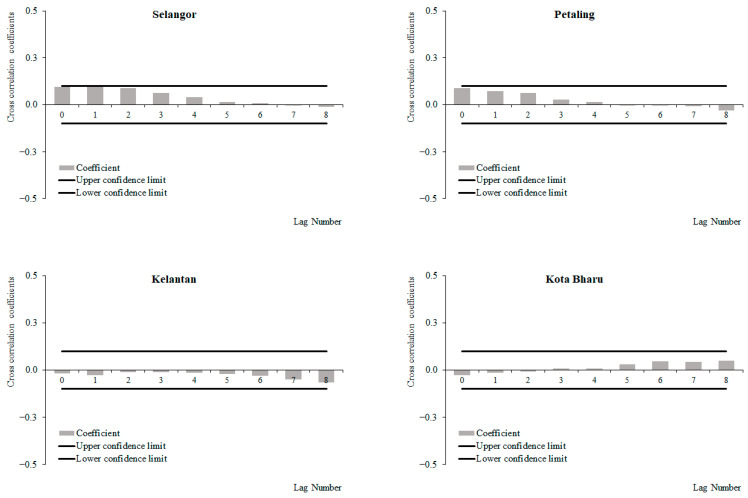
Cross-correlation function between dengue cases and wind speed in Selangor, Petaling, Kelantan and Kota Baharu, 2011 to 2019.

**Figure 13:**
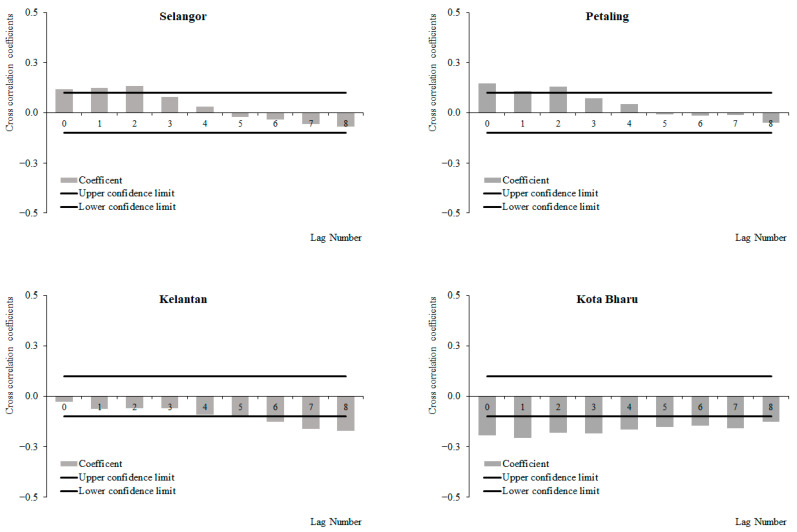
Cross-correlation function between dengue cases and wind speed in Selangor, Petaling, Kelantan and Kota Baharu, 2015 to 2019.

**Figure 14:**
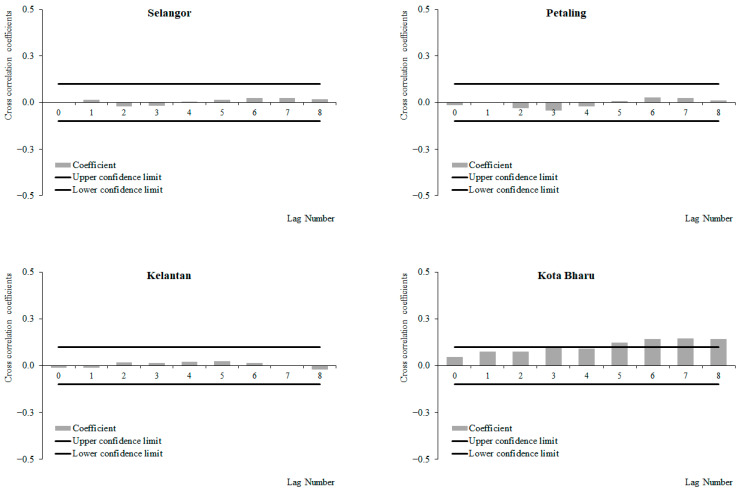
Cross-correlation function between dengue cases and wind speed in Selangor, Petaling, Kelantan and Kota Baharu, 2011 to 2014.

For the period between 2011 and 2019, a positive correlation between weekly dengue cases and wind speed at lag 0, which decreased with subsequent lags, was observed in Selangor and Petaling, respectively, while negative correlations between weekly dengue cases and wind speed at lag 0 were observed, which increased with subsequent lags in Kelantan and Kota Baharu, respectively (Figure 12). The highest correlation values were observed at lag 0 for Selangor (0.096), Petaling (0.088), and lag 8 for Kelantan (−0.065) and Kota Baharu (0.049), respectively. None of these correlations were significant. For the period from 2015 to 2019, a positive correlation between weekly dengue cases and wind speed was observed at lag 0, which decreased with subsequent lags with significant positive correlations at lag 0 onwards in Selangor and Petaling, respectively. Negative correlations between weekly dengue cases and wind speed were observed at lag 0, which increased with subsequent lags, and had significant negative correlations at lag 0 and 6 onwards for Kelantan and Kota Baharu (Figure 13). The highest correlation values were observed at lag 0 for Petaling (0.148), lag 1 for Kota Baharu (−0.206), lag 2 for Selangor (0.134) and lag 8 for Kelantan (−0.172). During the period between 2011 and 2014, no significant correlations were reported between weekly dengue cases and wind speed except for in Kota Baharu (Figure 14). Overall negative/reduced positive correlations were observed between weekly dengue cases and wind speed in all areas from 2011 to 2019 and 2015 to 2019, with significant correlations being observed for the period from 2015 to 2019.

Table 3 describes the cross correlation estimates between dengue cases and EO-based meteorological data, in Malaysia from 2011 to 2019.

### 3.3. Multivariate Linear Regression Analysis between Meteorological Variables and Dengue Cases

Following the multivariate linear regression analysis, rainfall, temperature and wind speed explained 48.2% of the variance of dengue cases in Selangor state. Rainfall (Beta = −0.806) contributed to the highest variance in dengue cases, followed by temperature (Beta = −0.409) and wind speed (Beta = 0.046). Both rainfall and temperature had significant effects on dengue cases as shown in Table 4.

## 4. Discussion

### 4.1. EO-Based and Ground Station Data Correlations

This study examined the correlation between satellite EO-based observations of temperature, wind speed and rainfall with ground station data. The analysis shows moderate to strong positive correlations (ρ = 0.519 to 0.838) that were statistically significant for all three variables between the data recorded by satellites and ground stations. The highest mean correlations were observed for temperature (mean ρ = 0.779), followed by rainfall (mean ρ = 0.687) and wind speed (mean ρ = 0.639). Similar findings were reported in previous studies with significant positive correlations ranging from 0.400 to 0.730 and 0.850 to 0.870 for temperature and rainfall [9,10,24,25,26]. These consistent findings across studies could be attributed to methodological similarities such as deriving EO weather data from satellites, spatially matching EO data with ground station data and using longer data points for the correlation analysis. This suggests that meteorological data derived from satellites are comparable to ground station data. In this study, the correlation between EO-based data derived from satellites with ground station data proves that the EO-based data correlates well to the ground station data. This correlation is valid as we used similar spatially matched ground stations and EO-based data which produced good correlations between the two observations. With recent technological advancements, satellites are able to provide meteorological data of higher spatial resolution and quality. Additionally, the use of a large time series data set which constitutes 9 years with 468 weekly data points in this study further increases the robustness of the correlation analysis and results. Therefore, meteorological data recorded from satellites can be used as a viable alternative to the conventionally used ground station data. In the next part of this study, having established strong correlations between satellite and ground station data, we used satellite-based data to analyse the correlation between dengue cases and meteorological variables. 

### 4.2. Correlation between EO-Derived Meteorological Data and Dengue Cases

This study found significant positive correlations between rainfall and dengue cases in Selangor and Petaling during the periods between 2011 to 2019, wherein an initial negative correlation between rainfall and dengue cases was observed from lag 0 to 1 weeks, following which there was a positive correlation between rainfall and dengue cases. An increasing positive correlation was observed after a 2 to 3-week lag which was significant for lag 4 and 6 weeks onwards in Selangor and Petaling. The initial negative correlation between rainfall and dengue cases for lags 0 to 1 can be explained by the effect of excessive rainfall, causing flushing, overflowing, the disruption of mosquito breeding sites, and destruction to the developing larvae [27]. Following this, an increasing pattern of positive correlations between rainfall and dengue cases with subsequent lags (weeks) was observed. Post rainfall conditions become more favourable for mosquito breeding sites [19]. The time from hatching to the development of an adult Aedes mosquito is ~2 weeks [28]. The extrinsic and intrinsic incubation period of the virus is ~12 and 10 days, respectively [29,30]. This duration fits well with the findings of this study, which reveal that from the onset of rainfall, it may take from 1 to 6 weeks before an increase in dengue activity is observed. Similar findings were observed during the periods between 2015 to 2019, wherein positive significant correlations between rainfall and dengue cases were reported at lags 2, 4, 5, and 7 onwards for Selangor, Petaling, Kelantan and Kota Baharu, respectively. Similarly, studies conducted in Myanmar, Thailand and Malaysia all reported that rainfall is an important factor that regulates the abundance of outdoor dengue mosquito breeding, whilst dengue outbreaks are commonly observed following a rainy season [30,31,32]. This is because rainfall might exert its effect on dengue infection partly through the creation of more breeding sites [33,34]. In this study, the east coast region of Peninsular Malaysia (i.e., Kelantan and Kota Baharu) reported no obvious and negative correlation patterns between rainfall and dengue cases for the time period from 2011 to 2019 and 2011 to 2014, respectively. Several reasons could explain these findings. The presence of non-seasonal patterns of dengue cases in Kelantan and Kota Baharu during the periods from 2011 to 2014 could have obscured the correlations between rainfall and dengue cases in these areas (Appendix A—Figure A3). Unlike in Kelantan and Kota Baharu, dengue cases in Selangor and Petaling displayed a seasonal pattern which was observed from 2011 onwards (Appendix A—Figure A4). In addition, changes in the surveillance and testing process for dengue cases, which occurred from 2014 to 2015, could have also impacted the correlation in these areas, where prior to this change, the number of dengue case reported in Kelantan and Kota Baharu were much lower compared to Selangor and Petaling. Furthermore, as Selangor and Petaling are highly urbanized and populous areas compared to Kelantan and Kota Baharu, the risk of dengue transmission increases and an increase the number of individuals being tested for dengue infections would be detectable, thereby, increasing the detection rate and number of dengue cases in Selangor and Petaling.

Our results also show a significant positive correlation between temperature and dengue cases observed in the east coast region of Peninsular Malaysia (i.e., Kelantan and Kota Baharu) throughout the time periods (2011 to 2019, 2011 to 2014 and 2015 to 2019). In addition, positive correlations between temperature and dengue cases in Kelantan and Kota Baharu were observed almost immediately (lag 0 to 3 weeks) and were significant at lags ranging from 0 to 6 weeks throughout all the time periods. The almost instant effect of temperature on the increase in dengue cases can be attributed to warm temperatures leading to an increase in dengue transmission due to increased mosquito populations [30]. We also found significant positive correlations with increased lags between temperature and dengue cases in Kelantan and Kota Baharu throughout all the time periods, while negative correlations with increasing lags were observed in Selangor and Petaling throughout all the time periods. Several reasons can explain this finding. In Peninsular Malaysia there are predominantly two monsoon seasons, the north east and south east monsoon, which affect the east (Kelantan and Kota Baharu) and west coast region (Selangor and Petaling), respectively. The north east monsoon (55% of total annual rainfall) results in heavier average rainfall compared to the south east monsoon (31% of total annual rainfall) [13]. As a result of this, the average temperatures in the north east (Kelantan and Kota Baharu) are generally lower compared to the west coast (Selangor and Petaling). Due to this, areas such as Kelantan and Kota Baharu might experience temperatures within the optimal temperature range (22 °C and 32 °C) for larvae development, mosquito longevity and fecundity [30]. In addition, an optimal temperature tends to result in increased mosquito feeding/breading rates and frequencies, a shorter extrinsic incubation period of the dengue virus, higher mosquito reproduction rates and accelerated viral replication, all of which would allow the Aedes mosquito to mature faster alongside higher survival rates [31]. It is important to note that while increases in temperature may promote mosquito growth, extreme temperatures may greatly affect this growth [32]. Studies have reported that prolonged high temperatures result in the downregulation of the heat shock protein gene within the Aedes mosquito, resulting in thermal stress and increased mortality [33]. Therefore, the negative correlation between temperature and dengue cases observed in this study could be attributed to the overall higher temperatures observed in the west coast region (Selangor and Petaling) compared to the east coast (Kelantan and Kota Baharu) [19,34]. Similar effects of temperature on dengue infections were reported in previous studies conducted in Malaysia [19], Vietnam [35] and Cambodia [36].

In this study, negative correlations were observed between weekly dengue cases and wind speed in all areas from 2011 to 2019 and 2015 to 2019, with significant correlations observed for the period from 2015 to 2019. During the period between 2015 and 2019, negative correlations between wind speed and dengue cases in Kelantan and Kota Baharu were observed almost immediately (lag 0) and were significant at lags ranging from 0 to 6 weeks, while reduced positive correlations between wind speed and dengue cases in Selangor and Petaling during the period between 2015 and 2019 were observed almost immediately (lag 0) and were significant at a lag of 0 weeks. Several reasons can explain the findings of this study. First, it was suggested that wind speeds of more than 5 knots (9.7 m/s) tend to suppress the Aedes mosquito flying activity which would affect their contact with humans and oviposition [3] and low wind speed promotes mosquitos’ rapid growth [3]. In addition, Malaysia, as a whole, generally experiences mean annual wind speeds of 1.8 m/s; however, there are some areas in the country that do experience strong winds during certain periods of the year [37]. For example, wind speeds during the southwest monsoon are often below 7 m/s, but during the northeast monsoon wind speeds could reach up to 15 m/s, particularly in the east coast of Peninsular Malaysia Kelantan and Kota Baharu [37]. Furthermore, strong wind speeds may also occur in certain areas in Malaysia as a result of typhoons from neighbouring countries [37]. The negative correlation observed in this study is in line with previous studies conducted in China [38], Sri Lanka [39] Malaysia [3] and Indonesia. 

EO-derived meteorological variables were able to explain almost 50% of the variation in dengue cases in Selangor. This finding indicates the considerable effect meteorological factors (i.e., rainfall, temperature and wind speed) have on dengue transmission. The balance unexplained variance in the dengue regression model could be due to the effect of other factors (i.e., host, vector, virus and environment) which were not accounted for in the model.

There are several strengths to this study, among which include the use of EO-data which was spatially matched with the ground station data when examining the correlation between meteorological data. This measure increased the accuracy and reliability the correlation analysis. Additionally, to date, this is the first study in Malaysia that utilized satellite EO meteorological data to examine the correlations between meteorological variables and dengue cases. The use of a longer study period (9 years) with several divisions of time lines for the analysis allowed for the study to account for the effects of changes in the dengue surveillance system. In addition, the selection of study sites at different areas within Peninsular Malaysia, allowed for the analysis to account for meteorological variations. The limitations of this study include the use of a low number of ground stations (four) for the correlation analysis. As dengue is a multifactorial disease, we acknowledge the possibility of the effects of other variables (i.e., Host, virus, vector and environment) on dengue transmission which could influence the findings of this study as these factors are often interrelated. However, the focus of this study was on examining the effects of meteorological variables derived from EO on dengue cases

## 5. Conclusions

Using EO data derived from satellites to correlate with dengue cases may not be a novel strategy from the global perspective; however, its use is novel in the Malaysian context, as this is the first study in the literature to have used Earth Observations satellite meteorological recordings to determine the correlation of meteorological variables and dengue cases. This study concludes that moderate to strong correlations were observed between meteorological variables recorded from EO data derived from satellites and ground stations, therefore justifying the use of EO data as a viable alternative to ground stations for recording meteorological variables. For the time period from 2015 to 2019, positive correlations were observed between weekly dengue cases with rainfall and temperature in all areas. During the same time period, wind speed only correlated positively with dengue cases in Selangor and Petaling. For the time period from 2011 to 2014, there were no significant correlations reported between weekly dengue cases with rainfall (in all areas) and wind speed (in all areas except Kota Baharu). However, a positive correlation between weekly dengue cases and temperature was reported in Kelantan and Kota Baharu. Evidence generated from this study could help in the management and control of dengue in Malaysia by developing early warning systems.

## Figures and Tables

**Table 1:** EO meteorological data.

Meteorological Data	Source	Measurement Unit	Resolution (Degrees, Longitude, Latitude)	Ground Area (km)
Rainfall	Global Precipitation Measurement (GPM)	Millimetres (mm)	0.10° × 0.10°	11.11 × 11.11
Temperature	Moderate Resolution Imaging Spectroradiometer (MODIS)	Celsius (°C)	0.05° × 0.05°	5.55 × 5.55
Wind speed	Climate Forecast System version 2 (CFSv2)	Meter per second (m/s)	0.205° × 0.205°	22.77 × 22.77

**Table 2:** Correlation between rainfall, temperature and wind speed data from EO and ground-based stations (*n* = 4), Malaysia (2011 to 2019).

Ground Station	Satellite Coordinate (Lat, Long)	Spearman Correlation	*p*-Value
**Rainfall**			
Kota Baharu *	6.17, 102.29	0.736	<0.001
Kuala Krai **	5.53, 102.20	0.669	<0.001
Subang ***	3.13, 101.55	0.702	<0.001
Sepang ****	2.74, 101.71	0.641	<0.001
**Temperature**			
Kota Baharu *	6.17, 102.29	0.838	<0.001
Kuala Krai **	5.53, 102.20	0.797	<0.001
Subang ***	3.13, 101.55	0.724	<0.001
Sepang ****	2.74, 101.71	0.757	<0.001
**Wind speed**			
Kota Baharu *	6.17, 102.29	0.742	<0.001
Subang ***	3.13, 101.55	0.658	<0.001
Sepang ****	2.74, 101.71	0.519	<0.001

Note: * Station ID 486150; ** Station ID 48616; *** Station ID 486470; **** Station ID 486500; Correlation analysis was conducted over a duration of 468 Weeks.

**Table 3:** Cross correlation estimates between dengue cases and EO-based meteorological data, Malaysia.

Dengue Cases and Rainfall				
Area/Duration	Highest Correlation	Lag	Direction of Correlation	Significance
**2011 to 2019**				
Selangor	0.223	8	Positive, increasing with increasing lags	Significant at lag 4 onwards
Petaling	0.155	8	Positive, increasing with increasing lags	Significant at lag 6 onwards
Kelantan	0.015	6	No obvious pattern	Non-significant
Kota Baharu	0.018	6	No obvious pattern	Non-significant
**2011 to 2014**				
Selangor	0.060	7	Positive with increasing lags	Non-significant
Petaling	0.045	3	Positive with increasing lags	Non-significant
Kelantan	−0.069	4	Negative with increasing lags	Non-significant
Kota Baharu	−0.024	0	Negative with increasing lags	Non-significant
**2015 to 2019**				
Selangor	0.304	8	Positive with increasing lags	Significant at lag 5 onwards
Petaling	0.181	8	Positive with increasing lags	Significant at lag 7 onwards
Kelantan	0.181	8	Positive with increasing lags	Significant at lag 4 onwards
Kota Baharu	0.142	6	Positive with increasing lags	Significant at lag 2 onwards
**Dengue Cases and Temperature**				
**Area/Duration**	**Highest** **Correlation**	**Lag**	**Direction of Correlation**	**Significance**
**2011 to 2019**				
Selangor	−0.090	8	Negative with increasing lags	Non-significant
Petaling	−0.217	8	Negative with increasing lags	Significant at lag 3
Kelantan	0.294	8	Positive with increasing lags	Significant at lag 2
Kota Baharu	0.227	8	Positive with increasing lags	Significant at lag 5
**2011 to 2014**				
Selangor	−0.01	7	Negative with increasing lags	Non-significant
Petaling	−0.240	4	Negative with increasing lags	Significant at lag 0
Kelantan	0.388	8	Positive with increasing lags	Significant at lag 0
Kota Baharu	0.346	8	Positive with increasing lags	Significant at lag 2
**2015 to 2019**				
Selangor	0.376	0	Positive, decreasing with increasing lags	Significant at lag 0
Petaling	0.511	0	Positive, decreasing with increasing lags	Significant at lag 0
Kelantan	0.247	8	Positive with increasing lags	Significant at lag 6
Kota Baharu	0.260	8	Positive with increasing lags	Significant at lag 5
**Dengue Cases and Wind Speed**				
**Area/Duration**	**Highest** **Correlation**	**Lag**	**Direction of Correlation**	**Significance**
**2011 to 2019**				
Selangor	0.096	0	Positive, decreasing with increasing lags	Non-significant
Petaling	0.088	0	Positive, decreasing with increasing lags	Non-significant
Kelantan	−0.065	8	Negative with increasing lags	Non-significant
Kota Baharu	0.049	8	Negative with increasing lags	Non-significant
**2011 to 2014**				
Selangor	0.025	7	No obvious pattern	Non-significant
Petaling	−0.042	3	No obvious pattern	Non-significant
Kelantan	0.022	4	No obvious pattern	Non-significant
Kota Baharu	0.146	7	Positive with increasing lags	Significant at lag 5
**2015 to 2019**				
Selangor	0.134	2	Positive, decreasing with increasing lags	Significant at lag 0
Petaling	0.148	0	Positive, decreasing with increasing lags	Significant at lag 0
Kelantan	−0.172	8	Negative with increasing lags	Significant at lag 6
Kota Baharu	−0.206	1	Negative with increasing lags	Significant at lag 0

**Table 4:** Multivariate Linear Regression analysis between meteorological variables and dengue cases in Selangor, Malaysia.

Model	Unstandardized Coefficients		Standardized Coefficients	t	Sig.
	B	Std. Error	Beta		
(Constant)	5.249	0.626		8.384	0.000
Selangor_Rain	−0.008	0.001	−0.806	−6.339	0.000 *
Selangor_Temp	−0.076	0.023	−0.409	−3.243	0.002 *
Selangor_Wind	0.014	0.037	0.046	0.389	0.699

Note. Predictors: (Constant), Temperature, rainfall and wind speed; Dependent; Dengue cases; * significant *p*-value (*p* < 0.05); Model Selangor R2 = 0.482; model significant (*p* < 0.001).

**Figure A1:**
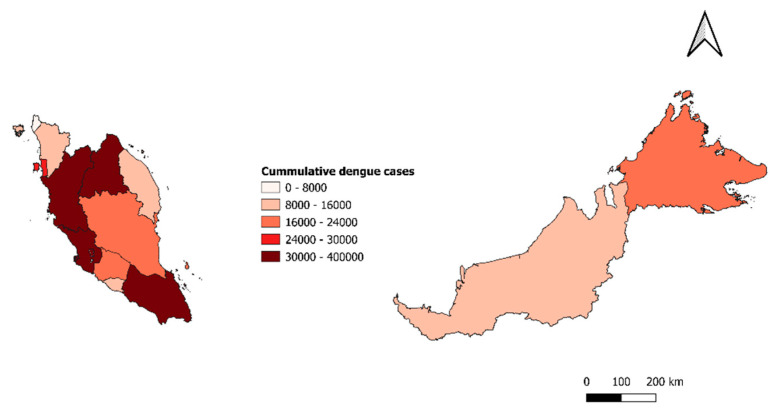
Cumulative dengue case by states, Malaysia 2011 to 2019.

**Figure A2:**
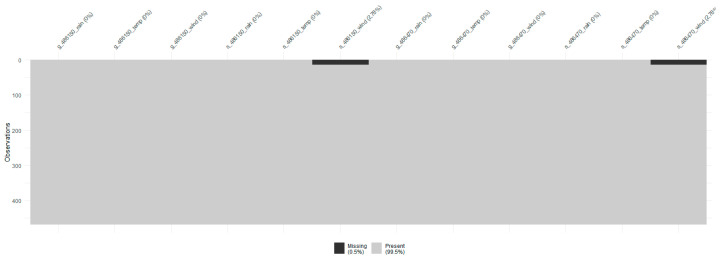
Missing data.

**Figure A3:**
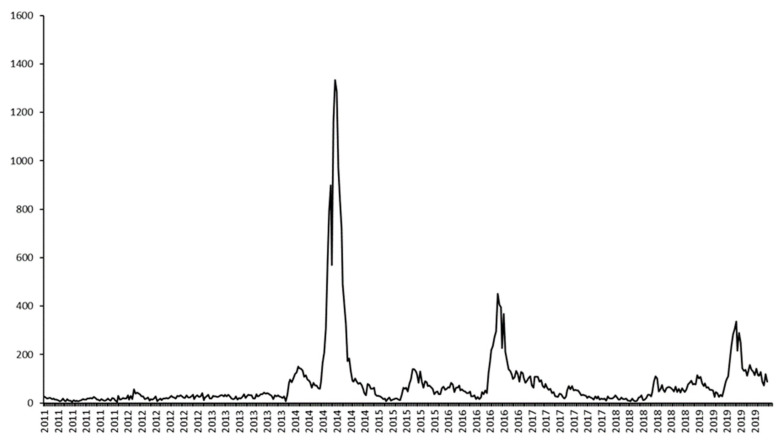
Dengue case in Kelantan, 2011 to 2019.

**Figure A4:**
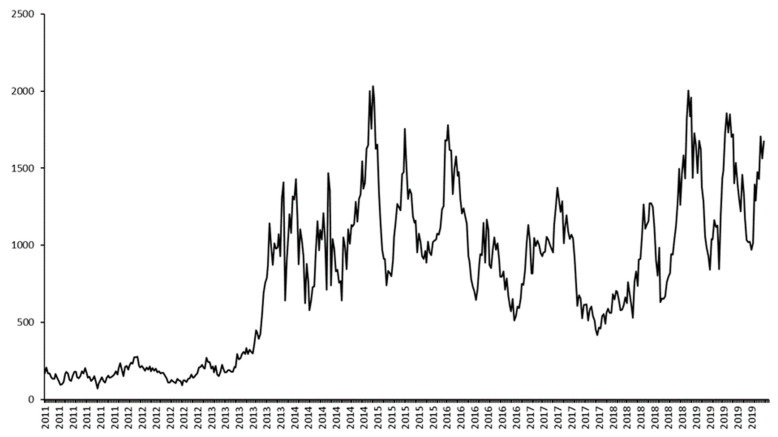
Dengue case in Selangor, 2011 to 2019.

**Figure A5:**
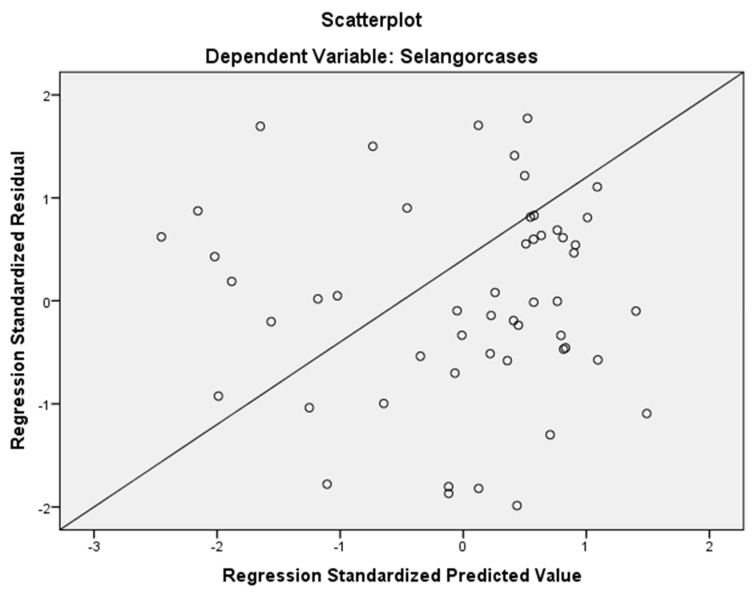
Scatter plot of predicted value and residuals.

## Data Availability

Data are available from authors upon request.
